# Traumatic cardiac arrest in Sweden 1990-2016 - a population-based national cohort study

**DOI:** 10.1186/s13049-018-0500-7

**Published:** 2018-04-23

**Authors:** T. Djarv, C. Axelsson, J. Herlitz, A. Stromsoe, J. Israelsson, A. Claesson

**Affiliations:** 1 Function of Emergency Medicine, Karolinska University Hospital, Stockholm, Sweden; 2 Karolinska Institutet, Department of Medicine Solna, Center for Resuscitation Science, Stockholm, Sweden; 3 The Prehospital Research Centre, University College of Borås, Borås, Sweden; 4 School of Health, Care and Social Sciences, Mälardalen University, SE-721 23 Västerås, Sweden; 5 Department of Internal Medicine, Division of Cardiology, Kalmar County Hospital, Kalmar, Sweden; 6 Kalmar Maritime Academy, Linnaeus University, Kalmar, Sweden; 7 Department of Medical and Health Sciences, Division of Nursing Science, Linköping University, Linköping, Sweden

**Keywords:** TCA, Prevalence, OHCA, Trauma, Resuscitation

## Abstract

**Background:**

Trauma is a main cause of death among young adults worldwide. Patients experiencing a traumatic cardiac arrest (TCA) certainly have a poor prognosis but population-based studies are sparse. Primarily to describe characteristics and 30-day survival following a TCA as compared with a medical out-of-hospital cardiac arrest (medical CA).

**Methods:**

A cohort study based on data from the nationwide, prospective population-based Swedish Registry for Cardiopulmonary Resuscitation (SRCR), a medical cardiac arrest registry, between 1990 and 2016. The definition of a TCA in the SRCR is a patient who is unresponsive with apnoea where cardiopulmonary resuscitation and/or defibrillation have been initiated and in whom the Emergency Medical Services (EMS, mainly a nurse-based system) reported trauma as the aetiology. Outcome was overall 30-day survival. Descriptive statistics as well as multivariable logistic regression models were used.

**Results:**

In all, between 1990 and 2016, 1774 (2.4%) cases had a TCA and 72,547 had a medical CA. Overall 30-day survival gradually increased over the years, and was 3.7% for TCAs compared to 8.2% following a medical CA (*p* < 0.01). Among TCAs, factors associated with a higher 30-day survival were bystander witnessed and having a shockable initial rhythm (adjusted OR 2.67, 95% C.I. 1.15–6.22 and OR 8.94 95% C.I. 4.27-18.69, respectively).

**Discussion:**

Association in registry-based studies do not imply causality but TCA had short time intervals in the chain of survival as well as high rates of bystander-CPR.

**Conclusion:**

In a medical CA registry like ours, prevalence of TCAs is low and survival is poor. Registries like ours might not capture the true incidence. However, many individuals do survive and resuscitation in TCAs should not be seen futile.

## What is already known on this subject?

- Traumatic cardiac arrest is rare and has a poor prognosis
- National population-based studies including aspects of importance for cardiac arrest and with complete outcome are sparse

## What this study adds?

- This national study shows that survival with a good neurological outcome is possible for patients suffering a TCA
- Survival after TCA has increased gradually over time and within the same range as survival after a medical CA

## Background

Out-of-hospital cardiac arrest (OHCA) is often fatal and affects some 300,000 people in Europe each year [1]. The majority of all reported cases are of a presumed cardiac aetiology [2–4], however non-cardiac OHCAs include cardiac arrest in a trauma patient [5, 6]. Trauma, resulting in cardiac arrest, is the main cause of death among young adults [7, 8]. There is no international consensus for a definition of traumatic cardiac arrest (TCA) but incidents traumatic in origin can clinically be diagnosed as cardiac arrests in the case of agonal or absent spontaneous respiration and absence of a central pulse [8, 9]. Resuscitation attempts including cardiopulmonary resuscitation (CPR) in TCAs have previously been considered futile and an inappropriate use of resources due to survival ratios as low as 0% and poor neurological outcome in survivors [7, 10, 11].

However, recent studies have presented survival ratios similar to OHCA linked to other aetiologies or even better, i.e. up to 17% [7–10, 12–17]. In addition, specific algorithms including one released by the European Resuscitation Council (ERC) has stressed the reversibility of aetiology behind the trauma [8, 9]. So far, large population-based studies on prevalence, prognostic factors of certain importance in cardiac arrests and outcomes are sparse for this relatively young group of patients.

Therefore, we conducted a national population-based cohort study with the primary aim of describing characteristics and 30-day survival following a TCA as compared with a medical out-of-hospital cardiac arrest (medical CA). The secondary aim was to describe factors associated with 30-day survival among TCA patients.

## Method

### Study design

This is a cohort study based on data from the Swedish Registry for Cardiopulmonary Resuscitation (SRCR) between 1990 and 2016. The SRCR is a nationwide population-based registry including OHCAs since 1990. From the start of the registry, the data for all Emergency Medical Service (EMS) managed OHCAs have been reported simultaneously, and according to Utstein methodology [18]. Since 2007, the SRCR has been web-based, today incorporating all EMS organisations in Sweden. The estimated coverage is close to 100% of all OHCAs since 2011(15). Further details have been described elsewhere [18].

### Settings

Sweden has a population of approximately 9,700,000 people within an area of 447 435km^2^. The EMS consisted in 2011 of 849 ambulances in 21 counties dispatched by 15 national dispatch centres reached by people in Sweden by one telephone number, 112. The call to the dispatch centre is free of charge, and both the use of EMS and acute care comes with a maximum fee of approximately 200 Euro per year for all inhabitants in Sweden. The ambulances are manned with two personnel each; in the majority of cases two registered nurses with additional paramedic training, all educated in advanced life support. Further, since 2005 according to a new law, all Swedish ambulances are staffed with at least one RN with special education in pre-hospital care. When responding to an OHCA, a dual-dispatch of two EMS units is used for quantitative reasons, providing advanced life support.

### Ethics approval and consent to participate

This was a study based on register data. Survivors are informed about their participation and can afterwards withdraw their inclusion in the registry. Since the start of the registry in 1990 only a handful of patients have withdrawn their participation. Deceased patients are included without consent. The Regional Ethical Review Board in Stockholm, Sweden approved the study, Dnr 2013/1959-31/4.

The manuscript does not contain individual person’s data, therefore consent for publications is not applicable.

### Participants and definitions

The definition of OHCA in SRCR is, a patient who is unresponsive where cardiopulmonary resuscitation including external chest compressions with or without ventilation (CPR) and/or defibrillation have been either initiated by a first responder and continued by the EMS or initiated by the EMS [18]. In close connection to the resuscitation attempt, the EMS report presumed aetiology of the cardiac arrest. The aetiology is based on the EMS crew’s best assessment made by synthesising the chain of events in the following categories: heart disease, pulmonary disease, trauma, choking, drowning, intoxication by prescribed drugs, suicide, sudden infant death syndrome or other. Therefore, for the purposes of this study, TCA was defined as an OHCA [18] with traumatic aetiology which, according to Utstein, is defined as “cardiac arrest directly caused by blunt, penetrating or burn injury”. Therefore, only OHCAs with the EMS crew’s best assessment as “trauma” were included, i.e. hypoxic cases such as hanging and drowning were excluded. Medical CA was used as a comparator group and included all the remaining cases with the exception of choking, drowning, suicide and drug overdose. All cases of TCA occurring between January 1st 1990 and December 31st 2016 were eligible for inclusion in the study and identified through the SRCR [18]. No further exclusion criteria were applied. In the case of multiple CAs within the same pre-hospital episode, only the first event was included.

### Data collection and categorisation

Patients were identified through the SRCR report sheet [19], where data on the following variables were collected: sex, age (in years), witnessed (yes by crew, yes by bystander or no), CPR before arrival of the EMS (yes or no), first documented heart rhythm (shockable (–Ventricle takycardia/Ventricle fibrillation) or non-shockable (Pulseless electric activity /asystole), place of OHCA (home, public or other), time between collapse and call (in minutes), time between collapse and start of CPR (in minutes), time between collapse and defibrillation (in minutes), time between collapse and arrival of the EMS (in minutes) as well as information on treatment provided during CPR (yes or no for the following: mechanical chest compressions, intubation, adrenaline, amiodarone; information on mechanical chest compression and amiodarone was available only in the web-based version). Information on the outcome, i.e. 30-day survival (yes or no) as well as information on Cerebral Performance Category (CPC) score at discharge from hospital among 30-day survivors was gathered from the SRCR and categorised as good (1-2) or poor (3-5) [19]. Data on CPC score was available only in the web-based version.

### Statistical analyses

**Fig. 1:**
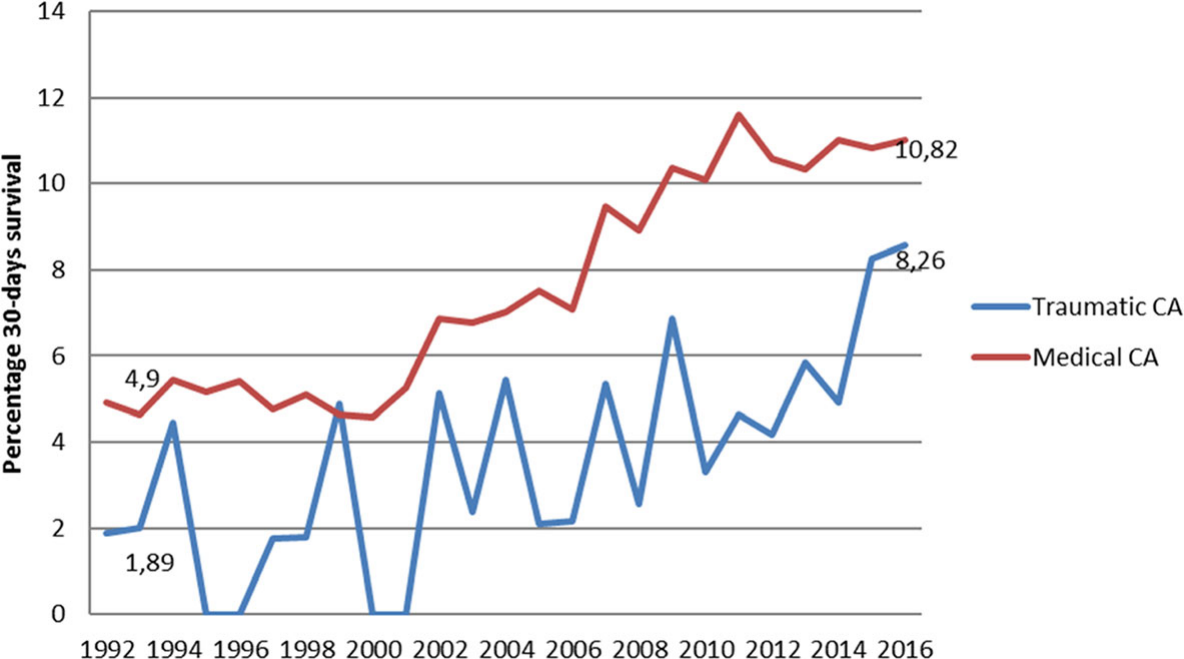
Survival ratio among 1774 TCAs compared to 72,547 medical OHCAs per year in the Swedish Cardiopulmonary Resuscitation Registry 1990-2016

**Fig. 2:**
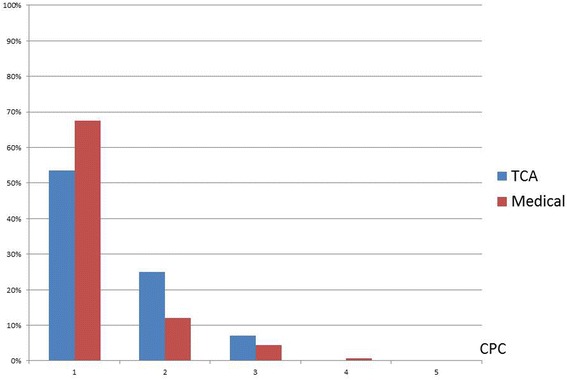
Cerebral Performance Category [19] at discharge from hospital among 28 Traumatic Cardiac Arrests (TCA) compared to 2644 medical out-of-hospital cardiac arrests in the Swedish Cardiopulmonary Resuscitation Registry 1990-2016

Descriptive statistics such as median with interquartile range (IQR) and mean with standard deviation (mean+/− SD) were used for patient characteristics. Characteristics of medical CA patients and TCA patients, as well as between survivors and non-survivors among TCAs were compared using Chi2 for dichotomous variables, and Student’s t-test for continuous variables. Two-sided tests were applied and a *p*-value ≤0.05 was interpreted as statistically significant. Missing data were kept missing, i.e. not imputed or estimated, and are presented in Table 1. Percentages in Table 2, Figs. 1 and 2 were based on numbers of patients with available information. A multivariable regression model was built based on significant variables, i.e. including witness status, shockable rhythm, given adrenaline, CPR before arrival of EMS, and time from call for help to arrival of EMS were used to estimate the associations between known prognostic factors and 30-day survival, expressed as odds ratios (OR) with 95% confidence intervals (95% CI). All statistical analyses were performed in SAS (version 9), SAS Inc., Cary, NC, USA.

**Table 1:** Characteristics of 72,547 medical out-of-hospital cardiac arrests (OHCA) compared to 1774 traumatic cardiac arrests (TCA) in the Swedish Registry of Cardiopulmonary Resuscitation between 1990 and 2016

	TCA Number (%) 1774 (100)	Medical OHCA Number (%) 72,547 (100)	*P*-value*
Sex			
Male	1385 (78)	49,940 (69)	< 0.01
Missing	45 (3)	1543 (2)	
Age			
Years, mean (SD)	51 (23)	70 (16)	< 0.01
Missing	281 (16)	2177 (3)	
Witnessed by			
Crew	253 (14)	10,277 (14)	0.34
Bystander	735 (41)	38,863 (54)	< 0.01
None	642 (36)	20,931 (29)	< 0.01
Missing for “None”	144 (8)	2476 (3)	< 0.01
CPR before arrival of EMS			
Yes	770 (56^a^)	31,563 (53^a^)	0.01
Missing	31 (2)	1142 (2)	
Initial rhythm			
Shockable	165 (9)	20,361 (28)	< 0.01
Missing	179 (10)	5924 (8)	< 0.01
Place of OHCA			
Public or other area	1440 (81)	24,278 (33)	< 0.01
Missing	15 (< 1)	454 (< 1)	
Time intervals in minutes (median)			
Collapse to call ^b^	2	3	< 0.01
Collapse to start of CPR ^b^	4	5	< 0.01
Collapse to defibrillation ^b^	12	12	0.82
Call to arrival of EMS	10	8	< 0.01
Treatment			
Adrenaline	1102 (62)	54,325 (75)	< 0.01
Missing adrenaline	12 (< 1)	442 (< 1)	
Intubation	812 (46)	32,728 (45)	0.73
Missing intubation	8 (< 1)	439 (< 1)	
Survival to 30 days	65 (4)	5941 (8)	< 0.01
Missing survival info	10 (1)	829 (1)	

^a^ Percentages based on numbers receiving CPR before EMS arrival /non crew-witnessed cases (TCA = 1377 and medical OHCA = 59,794)

^b^ Times only applicable to witnessed cases

*P-values were assessed with two-sided Chi^2^ test for dichotomous variables and Students t-test for continuous variables

**Table 2:** Differences in characteristics for 30-day survivors or not among 1764 prehospital traumatic cardiac arrests (TCA) in the Swedish Registry of Cardiopulmonary Resuscitation between 1990 and 2016

	30-day survivors 65 (100)	Non-survivors 1699 (100)	*P*-value*
Sex			
Male	52 (80)	1290 (76)	0.51
Age			
Years, mean (SD)	46 (21)	52 (33)	0.14
Witnessed by			
Crew	10 (15)	239 (14)	0.76
Bystander	39 (60)	671 (39)	< 0.01
None	9 (14)	609 (36)	0.01
CPR before arrival of EMS			
Yes	37 (57)	701 (41)	< 0.01
Initial rhythm			
Shockable	29 (44)	134 (8)	< 0.01
Place of OHCA			
Public or other area	56 (86)	1340 (79)	0.19
Time intervals in minutes (median)			
Collapse to call	1	2	0.03
Collapse to start of CPR	1,5	4	0.02
Collapse to defibrillation	8	13	0.05
Call to arrival of EMS	7	10	0.02
Treatment			
Adrenaline	28 (43)	1038 (61)	< 0.01
Intubation	21 (32)	768 (45)	0.05

*P-value assessed with two-sided Chi^2^ test or Students t-tes0

## Results

### Characteristics of traumatic cardiac arrests compared to medical cardiac arrests

In all, between 1990 and 2016 there were 1774 (2.4%) defined TCA cases within this study and 72,547 medical CA cases (Table 1). There were more men among TCA patients than among medical CA patients (78% vs 69%, respectively *p*-value < 0.01) and TCA patients were approximately 20 years younger than medical CA patients. TCAs were more likely not to have had anyone witness their arrest compared to medical CAs (36 and 29%, *p*-value < 0.01) but TCA patients received CPR before arrival of the EMS in 56% of cases versus 53% for medical CA, *p*-value 0.01). The heart rhythm was shockable in 10% of TCA cases compared to 30% of medical CA cases (*p*-value < 0.01). Delay from collapse to call, and from collapse to start of CPR were shorter among patients with witnessed TCA, whereas the opposite was found with regard to delay from call until arrival of the EMS, i.e. prolonged among patients with TCA.

### Survival

Overall, 30-day survival was 3.7% (65 patients) in TCA cases versus 8.1% (5941 patients) in medical CA cases (*p*-value < 0.01). The survival ratio gradually increased for both TCAs and medical CAs and more than doubled from 1990 to 2015 (Fig. 1). No significant difference was found between medical CA cases and TCAs found in a shockable rhythm (*p*-value 0.23, data not shown).

### Neurological outcome among survivors

A good neurological outcome, i.e. CPC 1-2, was found in 22 out of 28 (79%) patients with available information regarding survival after TCA as compared to 2380 out of 2644 (90%) with available information regarding survival after medical CA. However, the best CPC, i.e.1, was more common among medical CAs than TCAs (54% and 80% respectively, *p*-value < 0.01, Fig. 2).

### Prognostic factors for survival among TCAs

Patients surviving a TCA were more often witnessed by bystanders than non-survivors (*p*-value < 0.01, Table 2). Two main differences were found between survivors and non-survivors; survivors more often received CPR before EMS arrival (57% compared to 41%, *p*-value < 0.01, Table 2) and they also had a shockable initial rhythm more frequently (44% compared to 8%, *p*-value< 0.01, Table 2). Further, survivors had a significantly shorter delay from collapse to start of CPR, to defibrillation and from call to arrival of the EMS (Table 2). Regarding treatment, survivors significantly less often received adrenaline than non-survivors (43% and 61% respectively, *p*-value < 0.01).

### Association between prognostic factors and survival among TCAs

An independent association with increased survival to 30 days was found for bystander witnessed TCAs and a shockable rhythm, (adjusted OR 2.67, (95% C.I. 1.15 – 6.22) and OR 8.94 (4.27-19.67), respectively) while those given adrenaline had a poorer survival (adjusted OR 0.35, 95% C.I. 0.17 - 0.73, Table 3).

**Table 3:** Association between OHCA-variables and 30-day survival among 1764 TCAs in the Swedish Register for Cardiopulmonary Resuscitation between 1990 and 2016

OHCA variable	Crude OR (95% CI)	Adjusted OR (95% CI) ^a^
Bystander witnessed		
No	1.00 (Reference)	1.00 (Reference)
Yes	3.16. (1.39–7.17)	2.67 (1.15–6.22)
CPR before EMS arrival		
No	1.00 (Reference)	1.00 (Reference)
Yes	2.45 (1.13–5.27)	2.11 (0.92–4.84)
Initial rhythm		
Non-shockable	1.00 (Reference)	1.00 (Reference)
Shockable	9.79 (4.93–19.44)	8.94 (4.27–18.67)
Adrenaline		
No	1.00 (Reference)	1.00 (Reference)
Yes	0.48 (0.23–0.99)	0.35 (0.17–0.73)

^a^ Multivariable regression model including bystander witnessed or not, CPR before arrival of EMS, initial heart rhythm, adrenaline or not as well as time from call to arrival of EMS

## Discussion

This national population-based cohort study identifies poor overall 30-day survival of 3.7% for TCAs compared to 8.1% for medical CAs. However, survival for TCA gradually increased up to 8% in 2015 and shockable rhythm was an important prognostic factor. However, even though survival seems low, still 65 young individuals were successfully resuscitated with mostly good neurological outcome.

This study is an observational registry-based cohort study and one needs to keep in mind that association does not imply causality. However, the reason for the finding that survivors had good neurological outcome might be that many TCAs were witnessed, there were relatively short time intervals within the chain of survival and there were equally high rates of bystander-CPR as for medical CAs.

The prevalence of TCAs in our study indicates that TCA is the most common non-medical etiology [20] among all OHCAs although our figures were only about half that of recently reported data from Australia [15]. When assessing prevalence in our study, we must stress that SRCR is primary a cardiac arrest registry and it is possible that only a fraction of all TCA in Sweden is included. However Beck et al. included only OHCAs from metropolitan Perth, and traumas fulfilling the inclusion criteria might be more common in metropolitan areas than in areas on a national level which include rural areas as well [17]. Still both the Australian and Asian studies as well as ours indicate how rare TCAs are, which emphasises the need for simple and clear guidelines. Potentially survivable trauma deaths are most often due to extensive hemorrhage [21] demanding different treatments than standard ALS. In addition, an algorithm for TCAs was introduced by the ERC in 2015 [6]. The algorithm might constitute a useful guideline for the EMS to follow when confronted with a TCA.

Another rough indication of the prevalence of TCAs receiving CPR could be estimated based on national data for deaths and OHCAs in Sweden. Namely, during 1992-2014 a total of 62,697 individuals aged 35-79 years were reported dead due to either suicide, intoxications or trauma in Sweden [22]. During the same period of time 4664 cases of OHCA due to TCA, intoxication, drowning or suicide [20] receiving CPR were reported to the SRCR. So even if it is not possible to distinguish who suffered a trauma or not it indicates that CPR was initiated in approximately 7% of similar cases. Reasons for the low number of initiated CPR incidents in similar cases might be related to injuries not compatible with life or a late identification of the victim. Reasons may also be related to the concept that resuscitation attempts have been seen as futile, especially in blunt traumas [7, 10, 11] These concepts and figures might still challenge the implementation of algorithms for TCAs in clinical practice.

The overall survival of 3.7% found within this study is fairly poor and a comparison to previous studies is difficult to make since the range varies between 0 and 17% [14, 15] mainly due to differences in inclusion criteria and the lack of a national and population-based approach.

In Sweden, the general survival ratio for OHCAs has more than doubled during the study period [20]. This is probably due to a greater awareness and knowledge in society as well as the optimisation of each link in the chain of survival, e.g. dispatch with earlier recognition of OHCA, a greater focus on recognising agonal breathing alongside dispatcher-assisted CPR as well as dual dispatch using the EMS and first responders such as the fire department or police [23, 24].

The population suffering a TCA in our study is comparable to those described in previous literature, .i.e. consisting mainly of young males [14, 15, 17]. However, characteristics related to cardiac arrest differ in several aspects which might explain our higher survival ratio than both the Qatari and the Australian ratio. Both the Qatari and the Australian study found a much lower proportion with a shockable rhythm both for TCAs and medical OHCAs, and TCAs were less likely to receive bystander-CPR than medical OHCAs [15].

Interestingly, in our study, patients with TCAs received CPR before the arrival of the EMS slightly more often than medical CAs. In general, ratios for CPR before the arrival of the EMS in Sweden are high [2], perhaps due to general awareness, a simplified CPR-training with shorter courses and one manikin per participant, thereby making these basic lifesaving skills more widely available in the community. Further, the introduction of Automated External Defibrillators (AEDs) and Public Access Defibrillation (PAD) programmes alongside general awareness in society have certainly played a role for CPR before the arrival of the EMS [25]. The specific reasons for our ratio of bystander-CPR in TCAs are unknown but it is possible that relatives and others involved in the same trauma act as resuscitators.

The only significant factors associated with survival were whether the case was bystander witnessed, whether the first recorded rhythm was shockable and whether the patient was treated with adrenaline. However, it is possible that in cases with shockable rhythm the cardiac arrest came first and the trauma was a result of it, for example a patient having chest pain or arrhythmia might have braked in the car and thereby reduced the trauma. Our material includes patients with a cardiac arrest and trauma no matter if the trauma caused the cardiac arrest or if the cardiac arrest caused the trauma. However, EMS crews synthesise the whole picture at the scene including known preceding signs and the type of accident when categorising the cardiac arrest’s most likely etiology, i.e. cardiac or traumatic.

Regarding the poorer outcome among those given adrenaline the same finding has previously been shown in a registry-based study [26] and needs to be scrutinised in the light of confounding. Likely, adrenaline can be seen as a proxy for a longer time to ROSC and/or arrival to hospital. However, such information is missing in the current registry but the findings demand future studies. Also, this study was observational and we need to bear in mind that associations do not imply causality.

The good neurological outcome among survivors of TCA in this study, even if survivors are few in number, is in line with previous studies [14, 15] and is worth highlighting while implementing new algorithms for TCAs in clinical practice. Except for the constant improvement in the chain of survival, it is important to bear in mind that during the study period, general care of trauma has improved both before and after arrival in hospital. Many bigger cities in Sweden have trauma centers with high competence around the clock [21] and a new algorithm for management of TCAs was released in 2015 by European Resuscitation Council [9] stressing that TCAs are a state with a very low output, requiring immediate advanced directed interventions such as thoracotomy and external compression to stop bleeding. However, during the studied period there was no specific algorithm for prehospital management of TCAs in Sweden and thoracotomy in the field is still rare. However general trauma care has improved significantly between 1990 and 2016. The strengths of this study include the national and population-based design as well as the almost complete inclusion and constant validation of variables in the registry.

Limitations of this study include the design of the registry as a cardiac arrest registry rather than TCA registry and thereby the lack of information about the type of trauma and the severity index as well as information on the specific interventions undertaken such as thoracotomy [13]. Further, cases of TCA who have not received resuscitation in the form of chest compressions and/or defibrillation have not been included in the registry, due to the registry’s specific inclusion criteria. In Sweden, only certain units staffed with doctors or ambulance helicopters perform thoracotomy, so likely it is an uncommon procedure. Further, the registry we used has a main focus on cardiac arrests, not trauma, therefore inclusion might be affected by selection bias, i.e. patients initially dispatched as traumatic in origin might have been missed. However, efforts are made on a routine basis to validate and to find missing cases in EMS case records and hospital journals. Finally, in some of the variables the proportion of patients with missing information was not minor, only 22 out of the 41 TCA-survivors since the start of CPC recording had a CPC score recorded. Therefore the results regarding neurological outcome should be interpreted with caution.

## Conclusion

In conclusion, TCA seem to be rare and come with poor prognosis in a cardiac arrest registry like ours. However, many affected individuals are young and survivors appear to have a similarly good neurological outcome as medical CAs. Therefore, resuscitation in TCAs should not be seen as futile, but rather an area considered for improvements, and implementations of recent studies and guidelines [9, 16] might have a great potential to save even more lives.

## Abbreviations

AED: Automated External Defibrillators
CA: cardiac arrest
CPC: Cerebral Performance Category
CPR: Cardio-Pulmonary Resuscitation
EMS: Emergency Medical Services
OHCA: Out of hospital cardiac arrest
PAD: Public Access Defibrillation
ROSC: Return of Spontaneous Circulation
SRCR: Swedish Registry for Cardio-Pulmonary Resuscitation
TCA: traumatic cardiac arrest
